# Radiomics based on ^18^F‐FDG PET/CT could differentiate breast carcinoma from breast lymphoma using machine‐learning approach: A preliminary study

**DOI:** 10.1002/cam4.2711

**Published:** 2019-11-25

**Authors:** Xuejin Ou, Jing Zhang, Jian Wang, Fuwen Pang, Yongsheng Wang, Xiawei Wei, Xuelei Ma

**Affiliations:** ^1^ Department of Biotherapy West China Hospital and State Key Laboratory of Biotherapy Sichuan University Chengdu PR China; ^2^ Department of Thoracic Oncology West China Hospital Sichuan University Chengdu PR China; ^3^ State Key Laboratory of Biotherapy and Cancer Center West China Hospital Sichuan University Chengdu PR China; ^4^ Department of Neurosurgery West China Hospital Sichuan University Chengdu PR China; ^5^ School of Computer Science Nanjing University of Science and Technology Nanjing PR China; ^6^ Department of Nuclear Medicine West China Hospital Sichuan University Chengdu P.R. China; ^7^ Laboratory of Aging Research and Nanotoxicology State Key Laboratory of Biotherapy National Clinical Research Center for Geriatrics West China Hospital Sichuan University Chengdu Sichuan PR China

**Keywords:** breast lymphoma, diagnosis, linear discriminant analysis, machine‐learning, radiomic

## Abstract

**Purpose:**

Our study assessed the ability ^18^F‐fluorodeoxyglucose (FDG) positron emission tomography (PET)/computed tomography (CT) radiomics to differentiate breast carcinoma from breast lymphoma using machine‐learning approach.

**Methods:**

Sixty‐five breast nodules from 44 patients diagnosed as breast carcinoma or breast lymphoma were included. Standardized uptake value (SUV) and radiomic features from CT and PET images were extracted using local image features extraction software. Six discriminative models including PETa (based on clinical, SUV and radiomic features from PET images), PETb (SUV and radiomic features from PET images), PETc (radiomic features only from PET images), CTa (clinical and radiomic features from CT images), CTb (radiomic features only from CT images), and SUV model were generated using least absolute shrinkage and selection operator method and linear discriminant analysis. The areas under the receiver operating characteristic curve (AUCs), accuracy, sensitivity, and specificity were calculated to evaluate the discriminative ability of these models.

**Results:**

PETa and CTa models showed the best ability to differentiation in training and validation group (AUCs of 0.867 and 0.806 for PETa model, AUCs of 0.891 and 0.759 for CTa model, respectively).

**Conclusion:**

Models based on clinical, SUV, and radiomic features of ^18^F‐FDG PET/CT images could accurately discriminate breast carcinoma from breast lymphoma.

## Graphical abstract

Our study demonstrated that radiomics of 18F‐fluorodeoxyglucose (FDG) PET/CT images showed a promising ability to discriminate breast carcinoma from breast lymphoma based on the machine‐learning approach. Furthermore, clinical characteristics were able to improve the potential of radiomic features in differential diagnosis.

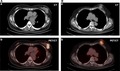

## 1 INTRODUCTION

Breast cancer is the leading cause of cancer‐related death among females worldwide which constitutes approximately 15% of all cancer‐related deaths in females.^1^ Standard treatment of breast cancer includes surgery, radiotherapy, chemotherapy, hormone therapy and targeted therapy. Most patients with early breast cancer receive surgical removal of tumors.^2^ Breast lymphoma, as a rare type of extranodal lymphoma, accounts for 0.5% of breast malignancies and 3% of extranodal lymphoma, with the predominant histopathology of diffuse large B‐cell lymphoma.^3^, ^4^ Although breast lymphoma is rare, its incidence has increased over the last four decades and will continually increase for younger women and for some subtypes.^5^ The clinical and imaging presentations of breast lymphoma mimic those of breast carcinoma, which often leads to misdiagnosis.^6^ Besides, the treatment of breast lymphoma is remarkably different from that of breast carcinoma. Chemotherapy and radiotherapy are principle treatment strategies for breast lymphoma with occasional need for surgical excision.^6^ Therefore, it is crucial to differentiate breast lymphoma from breast cancer to provide appropriate treatments for patients.

Mammography and ultrasonography (US) are commonly used imaging techniques in detecting breast lesions. However, it is quite hard to differentiate breast lymphoma from breast cancer based on mammographic or ultrasonographic imaging features, for they both showed unilateral, solitary, and a palpable mass.^7^ Fine‐needle aspiration and core needle biopsy are increasingly used in the diagnosis of lymphomas. It is important to have enough tissue in the biopsy sample for making accurate diagnosis. However, in most cases, needle biopsy samples are frequently limited in size, making the diagnosis challenging.^8^, ^9^, ^10^

In recent years, researchers found it feasible to differentiate lesions, which are hard to distinguish by conventional methods, using radiomic features and computer‐aided diagnosis systems. Texture analysis is an approach to characterize voxel‐intensity heterogeneity on different images.^11^ Texture analysis of computed tomography (CT) and magnetic resonance imaging (MRI) showed promising discriminative ability in various lesions, including breast lesions, lung lesions, and so on.^12^, ^13^, ^14^ Texture analysis on ^18^F‐FDG positron emission tomography (PET)/CT images also showed great diagnostic utility among lung lesions,^15^ renal lesions 16 and bone and soft‐tissue lesions.^11^ While the possibility using radiomic features to differentiate between carcinoma and lymphoma in the breast has not been investigated yet. Moreover, most studies only included radiomic features in their discriminative models. In clinical practice, patients characteristics such as age, sex, BMI, and so on, may provide additional diagnostic information when making differential diagnosis. Thus, the purpose of this study was to identify the ability of models generated using clinical characteristics, standardized uptake value (SUV) metrics, and ^18^F‐FDG PET/CT radiomic features to distinguish breast carcinoma from breast lymphoma.

## 2 MATERIALS AND METHODS

### 2.1 Study populations

**Figure 1:**
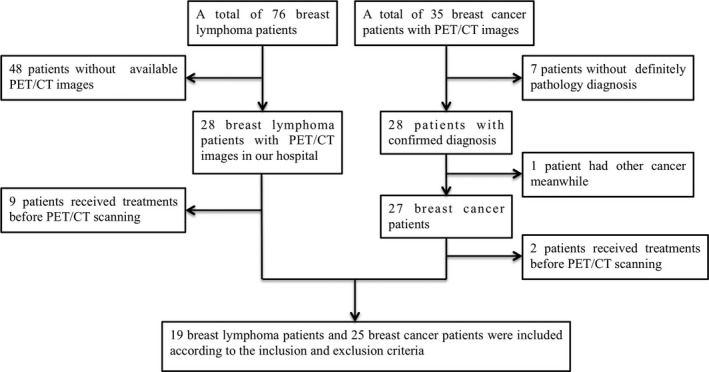
The workflow of patient selection. A total of 76 breast lymphoma patients and 35 breast cancer patients were enrolled in our study. According to our inclusion and exclusion criteria, 19 breast lymphoma patients, and 25 breast cancer patients were finally included

A total of 44 patients with breast cancer (25 patients) and breast lymphoma (19 patients), who received a basal ^18^F‐FDG PET/CT scanning in West China Hospital of Sichuan University from October 2013 to March 2018, were included in our single‐center retrospective study. Histopathological diagnosis was performed by biopsy or surgical resection. The inclusion criteria were as follows: (a) age ≥ 18 years old; and (b) the patients had conclusive histopathological diagnosis of breast cancer or breast lymphoma. The exclusion criteria included: (a) patients received treatments before ^18^F‐FDG PET/CT scans, including surgery, chemotherapy, and radiotherapy; (b) patients had other types of tumor besides breast carcinoma or breast lymphoma; (c) tumor size was not large enough for texture analysis (as local image features extraction (LIFEx) software calculated texture only for volumes of interest [VOIs] above 64 voxels). Details of patient selection were shown in Figure 1. Other basic characteristics of the patients were also extracted from our electronic medical record system. The study has been approved by the institutional review board Medical Ethics Committee, Sichuan University, and all patients signed an informed consent form. All procedures involving human participants were conducted in accordance with the ethical standards of the national research committee and with the 1964 Helsinki declaration and its later amendments.

### 2.2 ^18^F‐FDG PET/CT image acquisition and texture analysis

**Figure 2:**
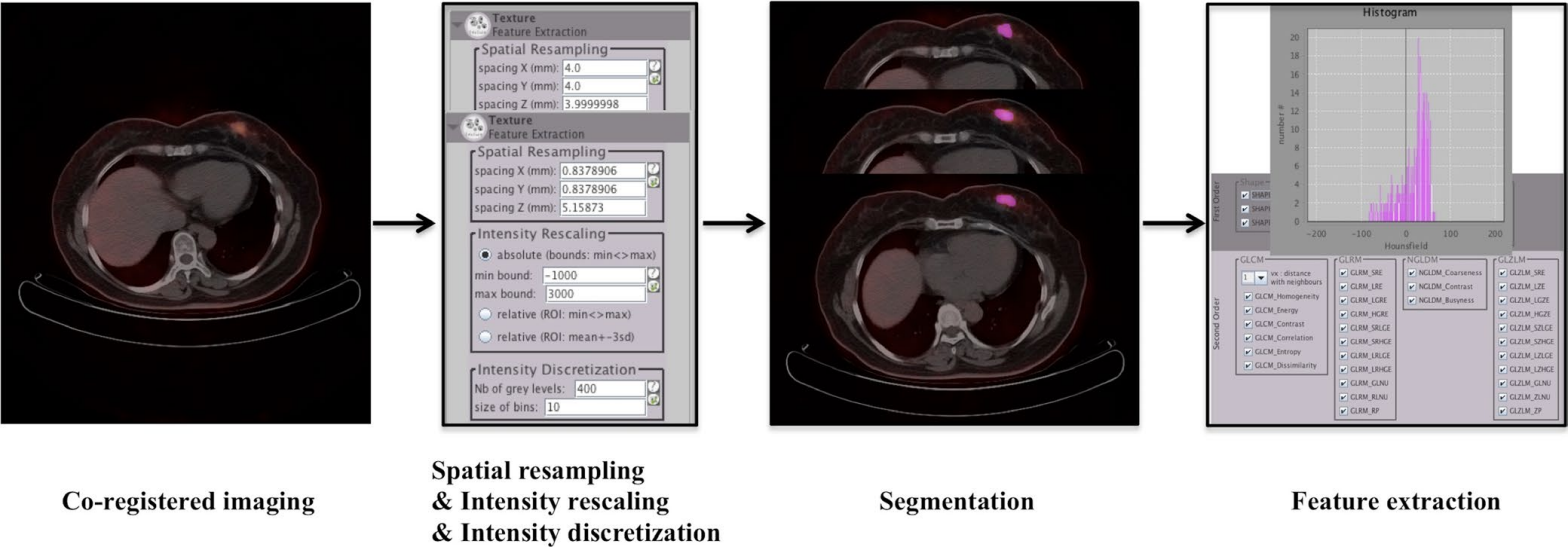
The flowchart of radiomics. After images were co‐registered, spatial resampling, intensity rescaling and intensity discretization was set automatically. Tumor segmentation was manually contoured in 3D VOI. Radiomic features from this volume were extracted, including first‐ and second‐order features

All the patients fasted for at least 6 hours before ^18^F‐FDG PET/CT scan. Blood glucose levels were controlled less than 128 mg/dL before intravenous injection of a 5.18‐MBq/kg dose of ^18^F‐FDG. Sixty minutes after injection, ^18^F‐FDG PET/CT images were acquired on a Gemini GXL PET/CT scanner equipped with a 16‐slice CT (Philips Medical System). Low‐radiation‐dose CT (5 mm slice thickness; tube voltage, 120 kV; tube current, 40 mAs) without intravenous contrast agent was performed from the head to the extremities for attenuation correction and anatomic localization. The radiomics workflow was shown in Figure 2. The LIFEx software (http://www.lifexsoft.org)^17^ was used for textural features analysis of the breast nodules on both PET and CT images within the same VOI at the same time. The region of interest was contoured manually on co‐registered images by an experienced radiologist who was blind to patients' clinical and pathological information. The whole layers in three‐dimensional VOI were delineated on all subsequent slices. For PET images, intensity discretization was automatically adjusted by the software with the number of gray levels of 64 bins, and intensity rescaling bounds were defined from 0 to 20. For CT images, intensity discretization was adjusted with the number of gray levels of 400 bins, and intensity rescaling bounds were from −1000 to 3000 HU. SUV metrics and radiomic features were all extracted using LIFEx software. The definitions and mathematical formulas of SUV metrics and radiomic features were summarized in Supporting Information S1.

### 2.3 Statistical analysis

Statistical analyses were performed on PYTHON software and IBM SPSS statistics software (version 25.0; SPSS Inc). Clinical parameters, SUV metrics, and radiomic features were analyzed in our study. Clinical parameters included age, tumor size, metastasis or dissemination and ^18^FDG dose. SUV metrics were SUVmin, SUVmax, SUVmean, SUVstd, SUVpeak, total lesion glycolysis (TLG), and metabolic tumor volume (MTV). Radiomic features including the first‐ and second‐order parameters were assessed in this study (summarized in the Table S1). Optimal features were selected using least absolute shrinkage and selection operator method, and then those selected features were fed into linear discriminant analysis (LDA) to generate discriminative models. Optimal features used in each model were shown in Table 1. A total of six discriminative models were generated: the PETa model was based on the clinical parameters, SUV metrics, and radiomic features extracted from PET images, the PETb model was the combination of SUV metrics and radiomic features from PET images, the PETc model was generated using radiomic features only from PET images, while the CTa model was constructed with clinical parameters and radiomic features from CT images, the CTb model was only based on radiomic features from CT images, and SUV model was established on SUV metrics. The datasets were divided into training and validation groups with the ratio of 4:1. The features and LDA functions were derived from the training cohort firstly and then genuinely applied to the independent validation cohort. In order to increase the robustness of these models, tenfold cross‐validation was performed in the analyzing process.

**Table 1:** Parameters used in each discriminative model

Method	PETa model	PETb model	PETc model	CTa model	CTb model	Conventional parameter
Parameters	Age CONVENTIONAL_TLG (mL) SHAPE_Volume (mL) GLCM_Contrast GLRLM_HGRE GLRLM_LRHGE GLRLM_RLNU GLZLM_LZE GLZLM_LZHGE	CONVENTIONAL_TLG (mL) SHAPE_Volume (# vx) GLRLM_HGRE GLRLM_LRHGE GLRLM_RLNU GLZLM_LZE GLZLM_HGZE GLZLM_SZHGE GLZLM_LZHGE	SHAPE_Volume (# vx) GLRLM_HGRE GLRLM_LRHGE GLZLM_LZE GLZLM_HGZE GLZLM_SZHGE GLZLM_LZHGE GLZLM_ZLNU	Age minValue maxValue HISTO_Kurtosis SHAPE_Volume (# vx) GLRLM_HGRE GLRLM_SRHGE GLRLM_LRHGE GLRLM_GLNU GLRLM_RLNU GLZLM_LZE GLZLM_HGZE GLZLM_SZHGE GLZLM_LZHGE GLZLM_ZLNU	minValue maxValue SHAPE_Volume (# vx) GLRLM_HGRE GLRLM_SRHGE GLRLM_LRHGE GLRLM_GLNU GLRLM_RLNU GLZLM_LZE GLZLM_HGZE GLZLM_SZHGE GLZLM_LZHGE GLZLM_ZLNU	TLG

Abbreviation: TLG, total lesion glycolysis.

A confusion matrix was determined using the data from histopathology and predictions of the LDA model. The areas under the receiver operating characteristic curve (AUC), accuracy, sensitivity, and specificity were calculated from the confusion matrix to evaluate the discriminative ability of these models. The differences of age, tumor size, stage, and injected ^18^FDG dose were compared using independent sample *t* test or Chi‐square test. A *P* value <.05 was considered significant.

## 3 RESULTS

Source data for open access, including clinical characteristics, SUV metrics and radiomic features, was included in the Supporting Information S2.

### 3.1 Patient characteristics

A total of 44 patients were included in this study. All patients were women and the median age were 52 (range, 26‐78) years old and 57 (range, 45‐80) years old respectively in lymphoma and carcinoma group. Of the 44 patients, 25 (25/44, 56.82%) had breast carcinoma, and 19 (19/44, 43.18%) had breast lymphoma. As texture of breast nodules and their relationship with pathologic diagnosis were what we analyzed, and the patient sample was quite small. In order to increase sample size, we included several nodules from the same patient. At last, 31 nodules from breast lymphoma patients and 34 nodules from breast carcinoma patients were included. The median lymphoma nodule size was 2.4 cm (range 1.3‐12.3 cm) and median carcinoma nodule size was 2.15 cm (range 0.6‐8.5 cm). Patients' baseline characteristics were summarized in Table 2. All nodules were randomly split into two groups.

**Table 2:** Patient and nodule characteristics

Characteristics	Lymphoma	Carcinoma	*P* value
Numbers of patients (%)	19 (43.18%)	25 (56.82%)	
Age (y) Median (range)	52 (26‐78)	57.5 (45‐80)	<.001
Stage I‐II (%)	8 (42.11%)	12 (48%)	.807
Stage III‐IV (%)	11 (57.89%)	13 (52%)	—
Distant metastasis/dissemination (%)	9 (47.37%)	11 (44%)	.413
^18^FDG dose (mCi) (median (range))	8.71 (6.17‐10.54)	9.2 (5.6‐11.04)	.290
Number of nodules (%)	31 (47.69%)	34 (52.31%)	—
Nodule size (median (range))	2.4 (1.3‐12.3)	2.15 (0.6‐8.5)	.022

Abbreviation: FDG, fluorodeoxyglucose.

### 3.2 Differential ability assessment

Among the models, CTa and PETa models both showed an excellent ability to discriminate between breast carcinoma and breast lymphoma. In the training group, CTa predictive model showed the best discriminative ability with AUC of 0.891, accuracy of 0.892, sensitivity of 0.915, and specificity of 0.873. After clinical parameter (age) was excluded from the CTa model, CTb was generated. As shown in Table 3, the AUC, accuracy, sensitivity, and specificity of the CTb predictive model decreased both in training and validation group. Meanwhile, a similar trend could be found in the PET dataset. The PETa models that are based on the clinical characteristic (age), SUV metric (TLG), and radiomic features from PET images, possessed excellent ability in distinguishing the two breast malignancies, especially in the validation group with the highest AUC of 0.806. However, performances of PETb and PETc models systematically declined after age and TLG were deleted from PETa dataset one by one. The single parameter‐TLG showed limited ability of discrimination with the AUC of 0.641 in the training group and AUC of 0.668 in validation group. Performances of all models in the validation group were inferior to those in the training group, but the behaviors of these models were consistent. (See also in Table 3).

**Table 3:** Results of the six discriminative models in distinguishing breast lymphoma from breast carcinoma in the training and validation group

Predictive models	Training						Validation						
	Confusion matrix		AUC	Accuracy	Sensitivity	Specificity	Confusion matrix		AUC	Accuracy	Sensitivity	Specificity	*P* value
PETa model	206	46	**0.867**	**0.869**	**0.811**	**0.904**	43	15	**0.806**	**0.808**	**0.806**	**0.842**	**<.0001**
	22	246					10	62					
PETb model	155	97	0.760	0.765	0.861	0.715	27	31	0.689	0.715	0.818	0.680	.014
	25	243					6	66					
PETc model	146	106	0.734	0.740	0.834	0.693	24	34	0.666	0.692	0.800	0.660	.056
	29	239					6	66					
CTa model	216	36	**0.891**	**0.892**	**0.915**	**0.873**	37	21	**0.759**	**0.769**	**0.804**	**0.750**	**<.0001**
	20	248					9	63					
CTb model	163	89	0.745	0.750	0.799	0.718	23	35	0.578	0.600	0.575	0.611	.093
	41	227					17	55					
SUV metric (TLG)	84	168	0.641	0.651	0.866	0.603	21	37	0.668	0.692	0.875	0.651	.004
	13	255					3	69					

Note

Bold indicates *P* value <.0001.

PETa model (*P* < .0001) refers to the diacriminative model constructed by using SUV metrics (*P* = .004), PET radiomic features and clinical parameters;

PETb model (*P* = .014) refers to the model generated by using SUV metrics and radiomic features;

PETc model (*P* = .056) refers to the model constructed by using radiomic features only;

CTa model (*P* < .0001) refers to the model including CT radiomic features and clinical parameters;

CTb model (*P* = .093) refers to the model built by using CT radiomic features only.

Abbreviation: AUC, areas under the receiver operating characteristic curve.

**Figure 3:**
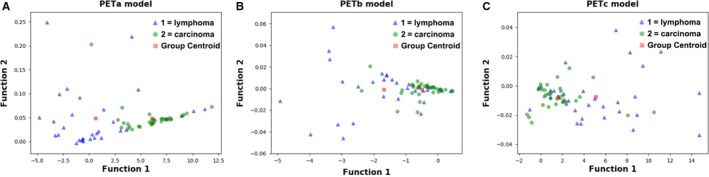
Relationship between the discriminant functions for breast lymphoma and breast carcinoma (triangles and circles) and for the group centroids (solid squares) in the PET models (PETa [A], PETb [B], and PETc [C] models ) in this study. The blue triangles refer to lymphoma, while the red circles stand for carcinoma. More overlapping could be seen in the PETb model and PETc model than in the PETa model. PET, positron emission tomography

**Figure 4:**
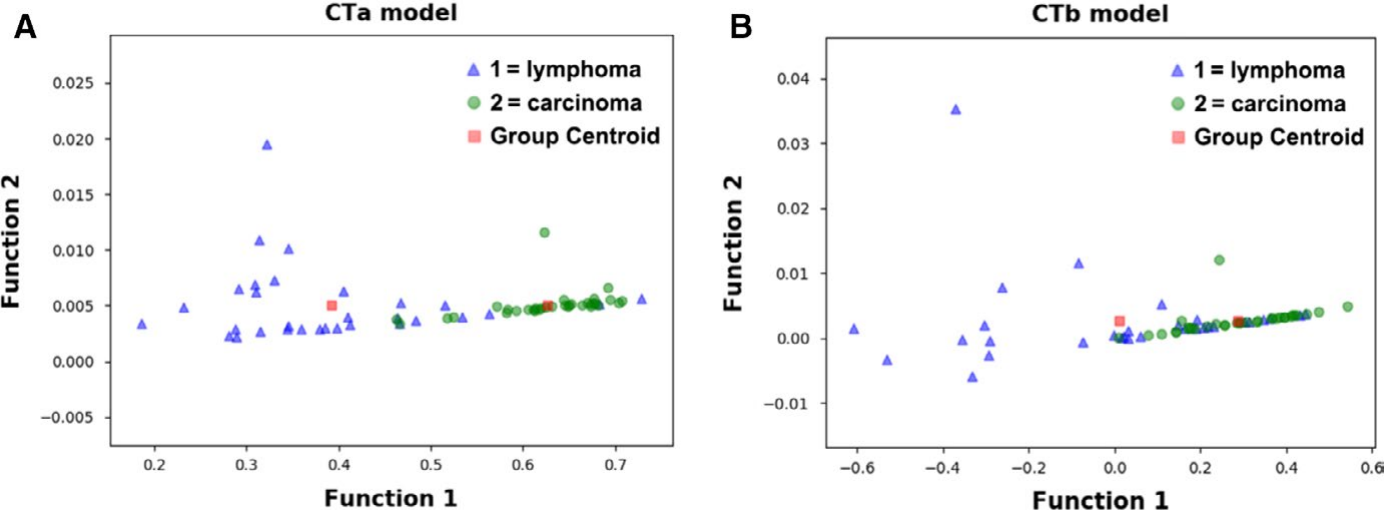
Relationship between the discriminant functions for breast lymphoma and breast carcinoma (triangles and circles) and for the group centroids (solid squares) in the CT models (CTa [A] and CTb [B] models) in the study. The blue triangles refer to lymphoma, while the red circles stand for carcinoma. Less overlapping was shown in the CTa model than in the CTb model. CT, computed tomography

Figure 3 showed the relationship between the canonical discriminative functions from PET models for the lymphoma and carcinoma groups (triangles and circles) and for the group centroids (squares). As only TLG was selected from the SUV metrics, figures reflecting the relationship between groups could not be generated in the SUV group. Qualitatively, analysis of the three discriminative models could separate breast carcinoma group from the breast lymphoma group. However, more overlapping was seen in the PETb and PETc models. The relationship between discriminative functions from CT models was displayed in Figure 4. A similar trend could be found in CT models. The CTa model showed a superior ability in separating the lymphoma group from the carcinoma group than the CTb model.

**Figure 5:**
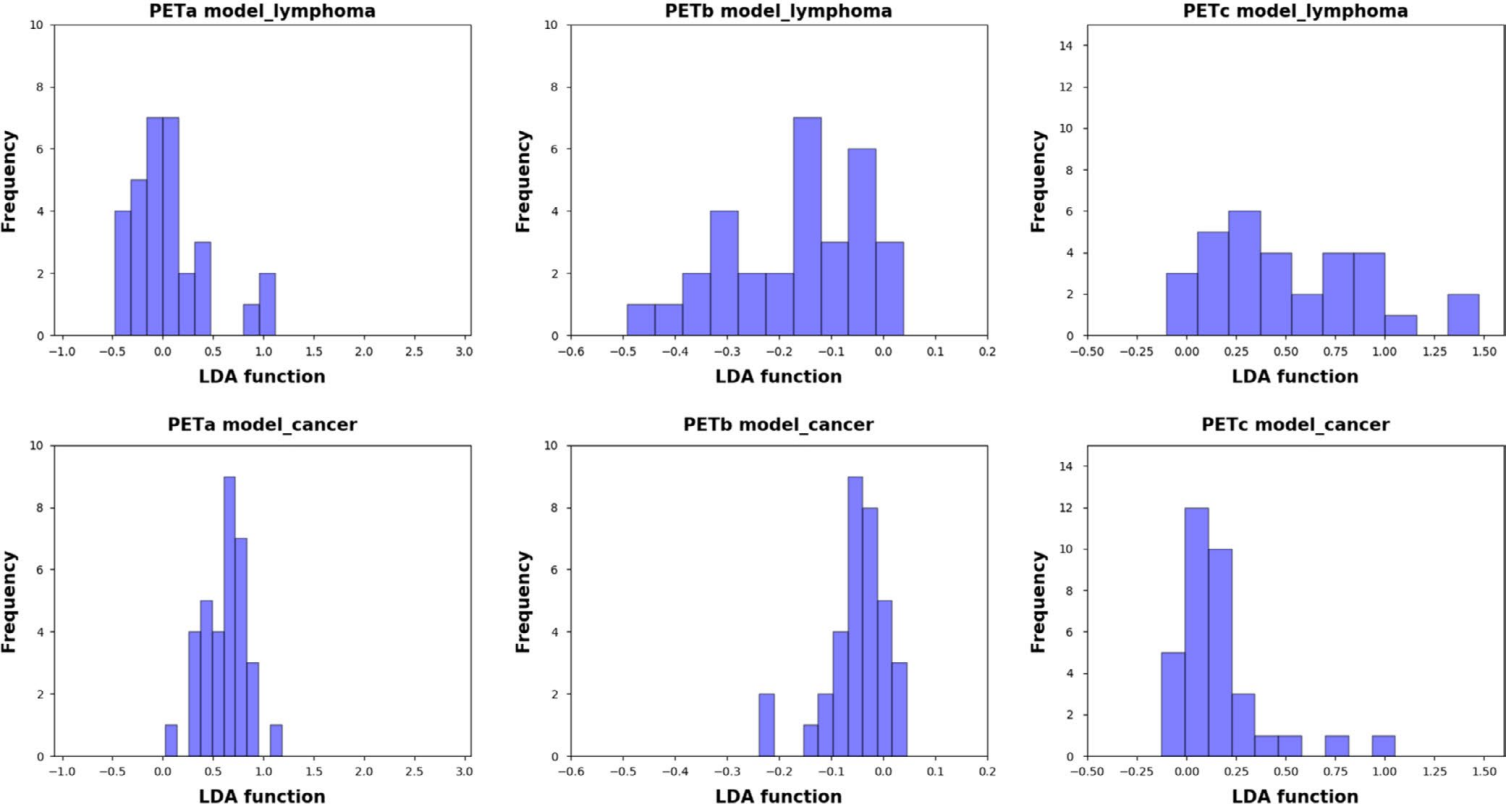
Examples of distributions of the LDA function determined for the breast cancer and breast lymphoma in the PET analysis for one cycle. Clear differences of LDA function value between breast lymphoma and breast carcinoma could be seen in the PETa model, while no significant shifts were noticed in the PETb and PETc model. LDA, linear discriminant analysis

**Figure 6:**
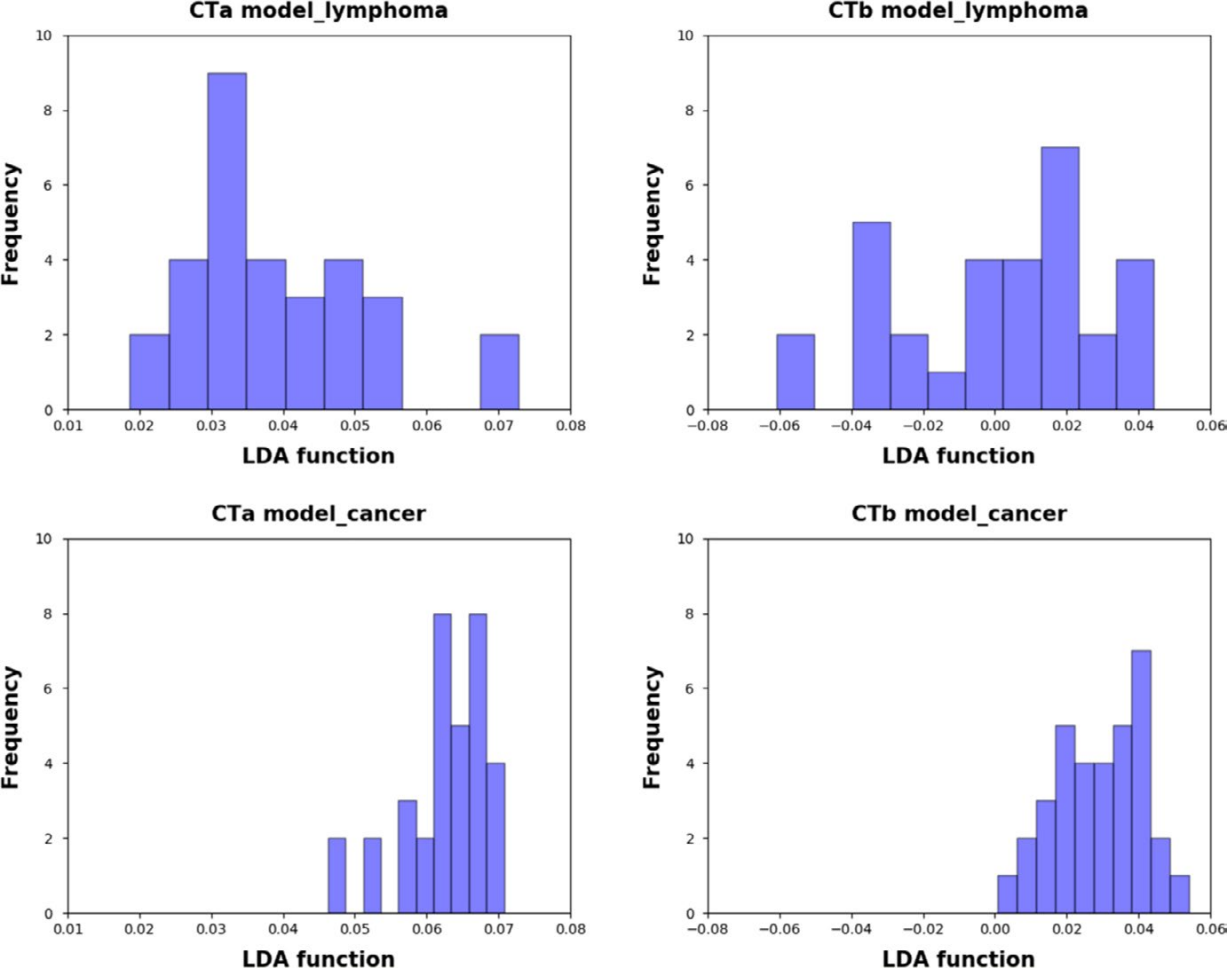
Examples of distributions of the LDA function determined for the breast cancer and breast lymphoma in the CT analysis for one cycle. Minimal overlapping was observed between breast lymphoma and breast carcinoma in the CTa model, while the LDA function value of breast lymphoma overlaps that of breast carcinoma a lot in the CTb model. CT, computed tomography; LDA, linear discriminant analysis

Figures 5 and 6 were examples of distribution of the LDA function determined for breast carcinoma and breast lymphoma for one cycle to illustrate the performance of LDA model. Analysis of PETa and CTa model both showed a clear shift of LDA function values between the two groups. In the PETa model, the LDA function values of lymphoma were mainly between −0.5 and 0.5, while the values of carcinoma were predominantly between 0.25 and 1.0. Similarly, in the CTa model, minimal overlaps were observed between the two groups. In contrast, in the analysis of PETb model, PETc model and CTb model, overlaps were obviously observed between the two groups.

**Figure 7:**
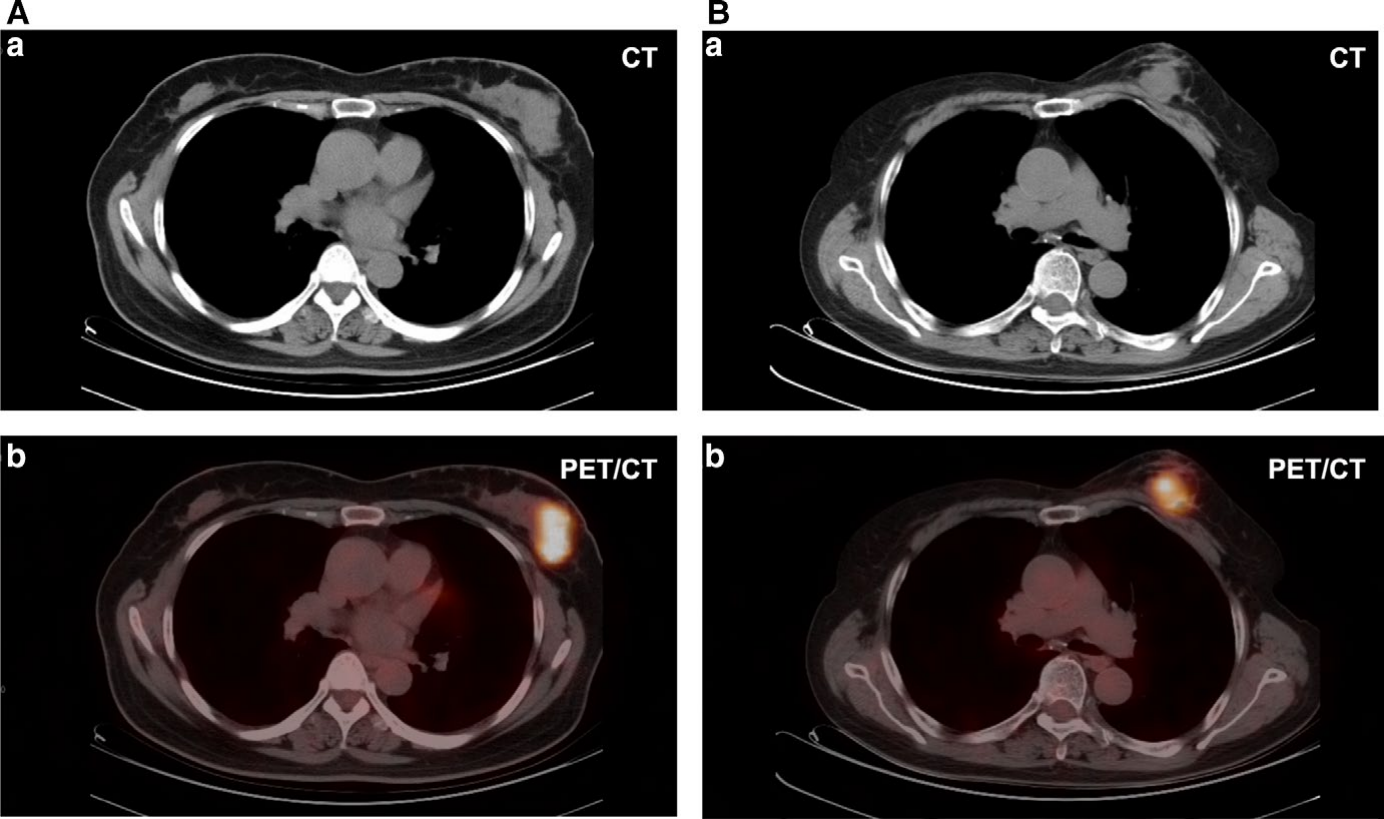
Two cases of CT and PET/CT images from patients with breast lymphoma (A) and breast carcinoma (B). CT, computed tomography; PET, positron emission tomography

Figure 7 showed two cases of PET/CT image from patients with breast lymphoma and breast carcinoma. Picture A: axial CT and PET/CT images (a and b, respectively) from a 52 year‐old women with breast lymphoma at the stage of IE (extranodal lymphoma at stage I). Tumor size is about 4 cm in diameter on the axial CT image. On the CT image, the tumor was a solitary lesion oval in shape with soft‐tissue density and an unclear edge. In the PET/CT image, the conventional SUV metrics were as follows: SUVmin = 0.88. SUVmean = 5.40, SUVstd = 2.75, SUVmax = 12.74, SUVpeak = 10.01, TLG = 160.93, MTV = 23ml. Picture B: axial CT and PET/CT images (a and b respectively) from a 68 year‐old women with breast carcinoma at stage I. The tumor size is about 3.1 cm on the axial CT image. On CT image, the lesion presented as a solitary mass round in shape, with soft‐tissue density, clear edge, and a regularly margin. In PET/CT image, the conventional SUV metrics are as follows: SUV_min_ = 1.20, SUV_mean_ = 5.17, SUV_std_ = 2.44, SUV_max_ = 13.02, SUV_peak_ = 9.98, TLG = 113.11, and MTV = 15 mL. As a result, we could not distinguish these two malignancies easily according to the conventional characteristics that we used quite a lot in clinical routine.

## 4 DISCUSSION

In this study, we conducted a preliminary research and assessed the ability of ^18^F‐FDG PET/CT radiomic features to differentiate breast carcinoma from breast lymphoma. We found that radiomics based on ^18^F‐FDG PET/CT could differentiate breast carcinoma from breast lymphoma using machine‐learning approach. What is more, clinical characteristics could help improve differential significance. To the best of our knowledge, this is the first report on the use of clinical parameters and radiomic features from ^18^FDG PET/CT images to differentiate between carcinoma and lymphoma in the breast.

In patients with breast nodules suspected of breast cancer, it is worthwhile to differentiate breast lymphoma from breast carcinoma since the therapeutic strategies and clinical outcomes are quite different.^6^ Biopsy is the golden standard for pathological classification, but it has several shortcomings: invasive, cannot be repeated, cannot provide whole body or spatial information, and limited tissue often makes the diagnosis difficult.^8^, ^9^, ^15^ Commonly used breast imaging techniques such as US, CT, mammography, and MRI are not efficient enough to distinguish them.^7^ For example, nine breast lymphoma patients in our study were excluded because they received breast surgery before PET/CT scanning as a result of misdiagnosis. Besides, the interpretation of images is observer‐dependent and relies heavily on individual experience.^15^ While radiomics, as a high‐throughput approach, could provide objective and quantitative assessment of medical images. Furthermore, Ultrasound, CT, mammography and MRI can only provide morphological and anatomical information of tumors, whereas, functional imaging PET/CT is able to reflect metabolic changes. Although ^18^F‐FDG PET/CT is not routinely recommended for diagnosis of breast mass according to the National Comprehensive Cancer Network guidelines,^17^ not only for its high cost but also for its limited sensitivity by missing small lesions (<5 mm),^18^ radiomics may make ^18^F‐FDG PET/CT a promising tool in the management of breast malignancies, by providing additional information such as tumor heterogeneity for assessment of tumor aggression.^19^

Radiomics‐enabled noninvasive evaluation provides multiple parameters. The use of radiomic features to distinguish different lesions has been reported in previous studies.^11^, ^12^ Chen et al^20^ demonstrated that texture parameters from FDG PET/CT images could help discriminate benign from malignant solitary pulmonary nodules. Miwa et al^21^ showed that FDG uptake heterogeneity assessed by fractal analysis could help to differentiate malignant from benign pulmonary nodules. Studies conducted by Feng et al suggested that texture analysis of CT images based on machine‐learning approach could identify small angiomyolipoma without visible fat from renal cell carcinoma.^22^ However, most previous studies focused on the differentiation of benign and malignant lesions. This may be due to great texture differences lying in the tissue complexity and heterogeneity between benign and malignant tumors. Recently, a study investigated the feasibility of tumor‐type prediction by analyzing brain metastatic lesions with radiomic features from MRI images.^23^ Their findings suggested that radiomics combined with machine learning classifier could provide high discriminative accuracy in the prediction of metastatic types in the brain.^23^ Another study conducted by Kirienko et al^15^ demonstrated that radiomics was able to distinguish primary lung carcinomas from metastatic lesions, even had the potential to identify lung cancer subtypes. Ha et al^24^ found that LDA with 24 radiomic features from FDG PET/CT images accurately clustered lung cancer histological subtypes of adenocarcinoma and squamous cell carcinoma. It has been widely realized that radiomics were promising in differentiating lesions, not only between benign and malignant lesions but also among malignancy subtypes. However, the possibility of pathological predictions of breast nodules with radiomic features from ^18^FDG PET/CT images was unclear. Thus, the results of our study support the potential of radiomic features as an imaging biomarker in the differential diagnosis of malignant nodules.

Besides, we found no studies, to our knowledge, had taken patients clinical characteristics such as sex, age, tumor size, distant metastasis, and so on, into consideration when constructing discriminative predicting models. Clinical characteristics, sometimes, may provide useful information in differential diagnosis. Our findings demonstrated clinical characteristics were able to improve the potential of radiomic features in identifying tumor lesions.

In this study, although discriminative ability of CTa model behaved best in the training group, its performance decreased and was inferior to that of PETa model in the validation group. Thus, we considered the behavior of CTa model as a slightly overfitting phenomenon. Therefore, in our perspective, the PETa model actually had a more stable and better discriminative ability than the CTa model. But some researchers have drawn different conclusions. Kirienko et al demonstrated that the predictive ability of texture analysis of CT images is better than that of PET images,^25^ but in their later study, they found that the discriminative ability of PET data is better.^15^ They explained that the latter study was evaluated in a larger cohort of patients, and they speculated that texture analysis of PET data could provide better tumor tissue characterization due to various reasons.^15^ For example, PET images could provide more complementary information than CT radiomic features since FDG uptake could reflect underlying biological differences.^26^ In our study, we also found that the discriminative ability of the combination of SUV metrics and PET radiomic features was slightly better than that of PET radiomic features and CT radiomic features. This suggested the important contribution of SUV metrics, specifically, the TLG. Total lesion glycolysis, as semiquantitative parameter of FDG PFT/CT, reflects glucose uptake within the whole tumor and evaluates tumor metabolic volume. TLG has also been proven to be highly correlated with Ki‐67 index which reflects cell proliferative activity.^27^ While, the cell proliferative activity of diffuse large B cell lymphoma and breast carcinoma are quite different, as diffuse large B cell lymphoma usually shows a much higher proliferative activity. This may be the possible reason why TLG could contribute to the differential diagnosis in this study.

In our study, ^18^F‐FDG PET/CT radiomics proved useful in the differential diagnosis of breast carcinoma and breast lymphoma, which might be due to the biological heterogeneity between the two malignant tumors. Tumor tissues and cells of breast carcinoma and breast lymphoma showed different characteristics in the aspect of necrosis, hemorrhage, proliferation, histological architecture, and so on.^28^, ^29^ As a result, the spatial FDG uptake distribution and tumor texture may be different on the PET/CT images. Radiomics provides us a way to quantify tumor heterogeneity objectively on medical images.^28^, ^29^ Unfortunately, no definitive correlations between each radiomic parameter and underlying tumor biological behaviors were investigated clearly. More research is needed for further exploration.

There are two main limitations in this study. Firstly, it is a retrospective single‐center study, which may result in patient selection bias. Secondly, the sample of this study was relatively small due to the low incidence of breast lymphoma. This, to some extent, may lead to potential overfitting in our preliminary results. Thus, future investigations with greater sample size are needed to verify our results.

## 5 CONCLUSION

Our study demonstrated that radiomics of ^18^F‐FDG PET/CT images showed promising ability to discriminate breast carcinoma from breast lymphoma based on machine‐learning approach. Furthermore, clinical characteristics were able to improve the potential of radiomic features in differential diagnosis. Future multi‐center studies with larger cohorts of patients are warranted to confirm our results to improve the differential diagnosis of breast lesions.

## Data Availability

Source data for open access, including clinical characteristics, SUV metrics and radiomic features, was included in the Supporting Information S2.
