# Supplementary material for: Radiomics based on 18F‐FDG PET/CT could differentiate breast carcinoma from breast lymphoma using machine‐learning approach: A preliminary study
Source: Cancer Med. 2019 Nov 25;9(2):496–506. doi: 10.1002/cam4.2711 (PMC6970046; doi:10.1002/cam4.2711)
Supplement: Supplementary file 1 [file CAM4-9-496-s001.docx]

**Supplementary material**

**Ability of 18F-FDG PET/CT radiomic features to distinguish breast carcinoma from breast lymphoma**

Supplementary Table1 Radiomic features extracted from PET and CT images, and Mathematical definitions for features assessed in the study.

| Conventional Indices | | First Order Features |
| --- | --- | --- |
| PET  CT | SUVmin  SUVmean  SUVstd  SUVmax  SUVpeak  TLG (mL)  minValue  meanValue  stdValue  maxValue | HISTO_Skewness  HISTO_Entropy_log_10_  HISTO_Entropy_log_2_  HISTO_Energy  SHAPE_Sphericity  SHAPE_Compacity  SHAPE_Volume (mL)  SHAPE_Volume(#vx) |
| Second Order Features | | |
| Grey-level co-occurrence matrix  (GLCM) | | **Neighbourhood grey-level different matrix (NGLDM)** |
| GLCM_Homogeneity  GLCM_Energy  GLCM_Contrast  GLCM_Correlation  GLCM_Entropy_log_10_  GLCM_Entropy_log_2_  GLCM_Dissimilarity | | NGLDM_Coarseness  NGLDM_Contrast  NGLDM_Busyness |
| Grey-level run-length matrix  (GLRLM) | | **Grey-level zone-length matrix**  **(GLZLM)** |
| Short-run emphasis (SRE)  Long-run emphasis (LRE)  Low grey-level run emphasis (LGRE)  High grey-level run emphasis (HGRE)  Short-run low grey-level emphasis (SRLGE)  Short-run high grey-level emphasis (SRHGE)  Long-run low grey-level emphasis (LRLGE)  Long-run high grey-level emphasis (LRHGE)  Grey-level non-uniformity for run(GLNU)  Run length non-uniformity(RLNU)  Run percentage (RP) | | Short-zone emphasis (SZE)  Long-zone emphasis (LZE)  Low grey-level zone emphasis (LGZE)  High grey-level zone emphasis (HGZE)  Short-zone low grey-level emphasis (SZLGE)  Short-zone high grey-level emphasis (SZHGE)  Long-zone low grey-level emphasis (LZLGE)  Long-zone high grey-level emphasis (LZHGE)  Grey-level non-uniformity for zone (GLNU)  Zone length non-uniformity (ZLNU)  Zone percentage (ZP) |

Abbreviation: SUV, Standardized Uptake Value; TLG: Total Lesion Glycolysis; HISTO, Histogram.

**Mathematical definitions:**

**Conventional Indices:**

**SUVmin:** minimum Standardized Uptake Value (SUV) in the Volume of Interest.

SUVmin=${min}_{i}{SUV}_{i}$

**SUVmean:** average SUV in the Volume of Interest.

SUVmean=$\frac{1}{N}\sum_{i} SUVi$

**SUVmax:** maximum SUV in the Volume of Interest.

SUVmax=${max}_{i}{SUV}_{i}$

**SUVpeak:** mean SUV in a sphere with a volume of ~0.5 or ~1 mL and located so that the average value in the VOI is maximum.

**TLG (mL):** Total Lesion Glycolysis is the product of SUVmean by Volume in mL [LARSON 1999]. SUVmean=V$\cdot\frac{1}{N}\sum_{i} {SUV}_{i}$

**First Order Features - Histogram**

**HISTO_Skewness**: asymmetry of the grey-level distribution in the histogram.

HISTO_Skewness:

$$\frac{\frac{1}{E}\sum_{i} {（HISTO(i)-\bar{HISTO})}^{3}}{{(\sqrt{\frac{1}{E}\sum_{i} {（HISTO(i)-\bar{HISTO})}^{2}})}^{3}}$$

HISTO（i）corresponds to the number of voxels with intensity i，E is the total number of voxels in the Volume of Interest and $\bar{HISTO}$ is the average of grey-levels in the histogram.

**HISTO_Entropy_log_10_**: randomness of the distribution.

HISTO_Entropy__log10_ =-$\sum_{i} p\left( i \right)\cdot log10 \left( p\left( i \right)+\varepsilon\right)$

Where p（i）is the probability of occurrence of voxels with intensity i and$\varepsilon$=2e-16

**HISTO_Entropy_log2**: the randomness of the distribution.

HISTO_Entropy__log2_ =-$\sum_{i} p\left( i \right)\cdot log2 \left( p\left( i \right)+\varepsilon\right)$

Where p（i）is the probability of occurrence of voxels with intensity i and$\varepsilon$=2e-16

**HISTO_Energy**: the uniformity of the distribution.

*HISTO*_*Energy*=${\sum_{i} p\left( i \right)}^{2}$

**First Order Features - SHAPE**

**SHAPE_Sphericity**: how spherical a Volume of Interest is. Sphericity is equal to 1 for a perfect sphere.

SHAPE_Sphericity= $\frac{\pi^{1/3}\cdot{(6V)}^{2/3}}{A}$

Where V and A correspond to the volume and the surface of the Volume Of Interest based on the Delaunay triangulation.

**SHAPE_Compacity**: how compact the Volume of Interest is.

SHAPE_Compacity= $\frac{A^{3/2}}{V}$

Where V and A correspond to the volume and the surface of the Volume Of Interest based on the Delaunay triangulation.

**SHAPE_Volume (mL and voxels):** the Volume of Interest in mL and in voxels.

SHAPE_Volume=$\sum_{i} Vi$

Where V*i* correspond to the volume of voxel *i* of the Volume Of Interest.

**Grey-Level Zone Length Matrix (GLZLM)**

The grey-level zone length matrix (GLZLM) provides information on the size of homogeneous zones for each grey-level in 3 dimensions. Element (i,j) of GLZLM corresponds to the number of homogeneous zones of j voxels with the intensity i in an image and is called GLZLM(i,j) thereafter.

**GLZLM_SZE, GLZLM_LZE**: the distribution of the short or the long homogeneous zones in an image.

GLZLM_SZE=$\frac{1}{H}\sum_{i} \sum_{j} \frac{GLZLM(i,j)}{j^{2}}$

GLZLM_LZE=$\frac{1}{H}\sum_{i} \sum_{j} GLZLM(i,j)\cdot j^{2}$

Where H corresponds to the number of homogeneous zones in the Volume of Interest.

**GLZLM_LGZE, GLZLM_HGZE:** the distribution of the low or high grey-level zones.

GLZLM_LGZE=$\frac{1}{H}\sum_{i} \sum_{j} \frac{GLZLM(i,j)}{i^{2}}$

GLZLM_HGZE=$\frac{1}{H}\sum_{i} \sum_{j} GLZLM(i,j)\cdot i^{2}$

**GLZLM_SZLGE, GLZLM_SZHGE**: the distribution of the short homogeneous zones with low or high grey-levels.

GLZLM_SZLGE=$\frac{1}{H}\sum_{i} \sum_{j} \frac{GLZLM(i,j)}{i^{2}j^{2}}$

GLZLM_SZHGE=$\frac{1}{H}\sum_{i} \sum_{j} \frac{GLZLM(i,j)\cdot i^{2}}{j^{2}}$

**GLZLM_LZLGE, GLZLM_LZHGE**: the distribution of the long homogeneous zones with low or high grey-levels.

GLZLM_LZLGE=$\frac{1}{H}\sum_{i} \sum_{j} \frac{GLZLM(i,j)\cdot j^{2}}{i^{2}}$

GLZLM_LZHGE=$\frac{1}{H}\sum_{i} \sum_{j} GLZLM(i,j)\cdot i^{2}\cdot j^{2}$

**GLZLM_GLNUz, GLZLM_ZLNU**: the non-uniformity of the grey-levels or the length of the homogeneous zones.

GLZLM_GLNUz=$\frac{1}{H}\sum_{i} {(\sum_{j} GLZLM(i,j))}^{2}$

GLZLM_ZLNU=$\frac{1}{H}\sum_{j} {(\sum_{i} GLZLM(i,j))}^{2}$

**GLZLM_ZP**: the homogeneity of the homogeneous zones.

GLZLM_ZP=$\frac{H}{\sum_{i} \sum_{j} (j\cdot GLZLM(i,j))}$

**Grey-Level Run Length Matrix (GLRLM)**

The grey-level run length matrix (GLRLM) gives the size of homogeneous runs for each grey level. This matrix is computed for the 13 different directions in 3D (4 in 2D) and for each of the 11 texture indices derived from this matrix, the 3D value is the average over the 13 directions in 3D (4 in 2D). The element (i,j) of GLRLM corresponds to the number of homogeneous runs of j voxels with intensity i in an image and is called GLRLM(i,j) thereafter.

**GLRLM_SRE, GLRLM_LRE**: the distribution of the short or the long homogeneous runs in an image.

GLRLM_SRE=Average over 13 directions $（\frac{1}{H}\sum_{i} \sum_{j} \frac{GLRLM(i,j)}{j^{2}}）$

GLRLM_LRE=Average over 13 directions $（\frac{1}{H}\sum_{i} \sum_{j} GLRLM(i,j)\cdot j^{2}）$

Where H corresponds to the number of homogeneous runs in the Volume of Interest.

**GLRLM_LGRE, GLRLM_HGRE**: the distribution of the low or high grey-level runs.

GLRLM_LGRE=Average over 13 directions $（\frac{1}{H}\sum_{i} \sum_{j} \frac{GLRLM(i,j)}{i^{2}}）$

*GLRLM*_*HGRE*=*Average* *over* 13 *directions （*$\frac{1}{H}\sum_{i} \sum_{j} GLRLM(i,j)\cdot i^{2}$*）*

**GLRLM_SRLGE, GLRLM_SRHGE**: the distribution of the short homogeneous runs with low or high grey-levels.

GLRLM_SRLGE=Average over 13 directions $（\frac{1}{H}\sum_{i} \sum_{j} \frac{GLRLM(i,j)}{i^{2}j^{2}}）$

GLRLM_SRHGE=Average over 13 directions （$\frac{1}{H}\sum_{i} \sum_{j} \frac{GLRLM(i,j)i^{2}}{j^{2}}$）

**GLRLM_LRLGE, GLRLM_LRHGE**: the distribution of the long homogeneous runs with low or high grey-levels.

GLRLM_LRLGE=Average over 13 directions $(\frac{1}{H}\sum_{i} \sum_{j} \frac{GLRLM(i,j)j^{2}}{i^{2}})$

GLRLM_LRHGE=Average over 13 directions （$\frac{1}{H}\sum_{i} \sum_{j} GLRLM(i,j)\cdot i^{2}j^{2}$）

**GLRLM_GLNUr, GLRLM_RLNU**: the non-uniformity of the grey-levels or the length of the homogeneous runs.

GLRLM_GLNUr=Average over 13 directions$\{\frac{1}{H}\sum_{i} {(\sum_{j} GLRLM(i,j))}^{2}$}

GLRLM_RLNU=Average over 13 directions $\{\frac{1}{H}\sum_{j} {(\sum_{i} GLRLM(i,j))}^{2}$}

**GLRLM_RP**: the homogeneity of the homogeneous runs.

GLRLM_RP=Average over 13 directions （$\frac{H}{\sum_{i} \sum_{j} （j\cdot GLRLM(i,j))}$）

**Neighborhood Grey-Level Different Matrix (NGLDM)**

The neighborhood grey-level different matrix (NGLDM) corresponds to the difference of grey-level between one voxel and its 26 neighbours in 3 dimensions (8 in 2D). An element (i,1) of NGLDM corresponds to the probability of occurrence of level i and an element (i,2) is equal to:

NGLDM（i, 2）=$\sum_{p} \sum_{q} \left\{ \begin{aligned} \left| \bar{M}（p,q）-i \right|\sim ifI(p,q=i) \\ 0\sim else \end{aligned} \right.$

where $\bar{M}$（p, q） is the average of intensities over the 26 neighbour voxels of voxel（p, q）.

**NGLDM_Coarseness**: the level of spatial rate of change in intensity.

NGLDM_Coarseness=$\frac{1}{\sum_{i} NGLDM(i,1)\cdot NGLDM(i,2)}$

**NGLDM_Contrast**: the intensity difference between neighbouring regions.

NGLDM_Contrast=$\left[ \sum_{i} \sum_{j} NGLDM(i,1)\cdot NGLDM(j,1)\cdot{(i-j)}^{2} \right]\cdot\frac{\sum_{i} NGLDM(i,2)}{E\cdot G\cdot(G-1)}$

where E corresponds to the number of voxels in the Volume of Interest and G the number of grey-levels.

**NGLDM_Busyness:** the spatial frequency of changes in intensity.

NGLDM_Busyness= $\frac{\sum_{i} NGLDM(i,1)\cdot NGLDM(i,2)}{\begin{matrix} \sum_{I} \sum_{J} \left| i\cdot NGLDM\left( i,1 \right)-j\cdot NGLDM\left( j,1 \right) \right| \\ with NGLDM(i,1)\neq0，NGLDM(j,1)\neq0 \end{matrix}}$

**grey level co-occurrence matrix (GLCM)**

The grey level co-occurrence matrix (GLCM) takes into account the arrangements of pairs of voxels to calculate textural indices. The GLCM is calculated from 13 different directions in 3D with aδ-voxel distance（$\left\| \vec{d} \right\|$ relationship between neighboured voxels. The index value is the average of the index over the 13 directions in space (X, Y, Z). Six textural indices can be computed from this matrix. An entry (i,j) of GLCM for one direction is equal to:

GLCM$\triangle x\triangle y(i,j)=\frac{1}{PairsROI}\sum_{p=1}^{N-\triangle x} \sum_{q=1}^{M-\triangle y} \left\{ \begin{matrix} 1 if(I(p,q)=i,I(p+\triangle x,q+\triangle y)=j \\ and I(p,q),I(p+\triangle x,q+\triangle y)\in ROI \\ 0 otherwise \end{matrix} \right.$

Where I(p,q) corresponds to voxel (p,q) in an image (I) of size N∗M. The vector $\overset{\to}{d}=(\triangle x,\triangle y)$covers the 4 directions (D1, D2, D3, D4, in 2D space or 13 directions (D1, D2, ..., D13, in 3D space and Pairs_ROI_.

The GLCM reflects the distribution of co-occurring pixel values at a given offset.

**GLCM_Homogeneity**: the homogeneity of grey-level voxel pairs.

GLCM_Homogeneity=Average over 13 directions ($\sum_{i} \sum_{j} \frac{GLCM(i,j)}{1+\left| i-j \right|}$)

**GLCM_Energy**: the uniformity of grey-level voxel pairs.

GLCM_Energy=Average over 13 directions ($\sum_{i} \sum_{j} GLCM{(i,j)}^{2}$)

**GLCM_Contrast**: the local variations in the GLCM.

GLCM_Contrast=Average over 13 directions ($\sum_{i} \sum_{j} {(i-j)}^{2}\cdot GLCM(i,j)$)

**GLCM_Correlation**: the linear dependency of grey-levels in GLCM.

GLCM_Correlation=Average over 13 directions ($\sum_{i} \sum_{j} \frac{（i-\mu_{i}）\cdot(j-\mu_{j})\cdot GLCM(i,j)}{\mathcal{o}_{i}\cdot\mathcal{o}_{j}}$)

where $\mu_{i}$ or $\mu_{j}$ corresponds to the average on row i or column j and $\mu_{i}$ and $\mu_{j}$correspond to the variance on row i or column j.

**GLCM_Entropy_log10**: the randomness of grey-level voxel pairs.

GLCM_Entropy_log10_=Average over 13 directions (-$\sum_{i} \sum_{j} GLCM(i,j)\cdot\log_{10} (GLCM(i,j)+\varepsilon)$)

Where ε= 2e-16

**GLCM_Entropy_log2**: the randomness of grey-level voxel pairs.

GLCM_Entropy_log2_=Average over 13 directions ( $\sum_{i} \sum_{j} GLCM(i,j)\cdot\log_{2} (GLCM(i,j)+\varepsilon)$)

where ε= 2e-16

**GLCM_Dissimilarity**: the variation of grey-level voxel pairs.

GLCM_Dissimilarity=Average over 13 directions ($\sum_{i} \sum_{j} \left| i-j \right|\cdot GLCM(i,j)$)

References:

Nioche C, Orlhac F, Boughdad S, Reuzé S, Goya-Outi J, Robert C, Pellot-Barakat C, Soussan M, Frouin F, Buvat I. LIFEx: A Freeware for Radiomic Feature Calculation in Multimodality Imaging to Accelerate Advances in the Characterization of Tumor Heterogeneity. Cancer Res. 2018 Aug 15; 78(16): 4786-4789.

Note: the mathematic formulas are downloaded from website: <http://www.lifexsoft.org>
